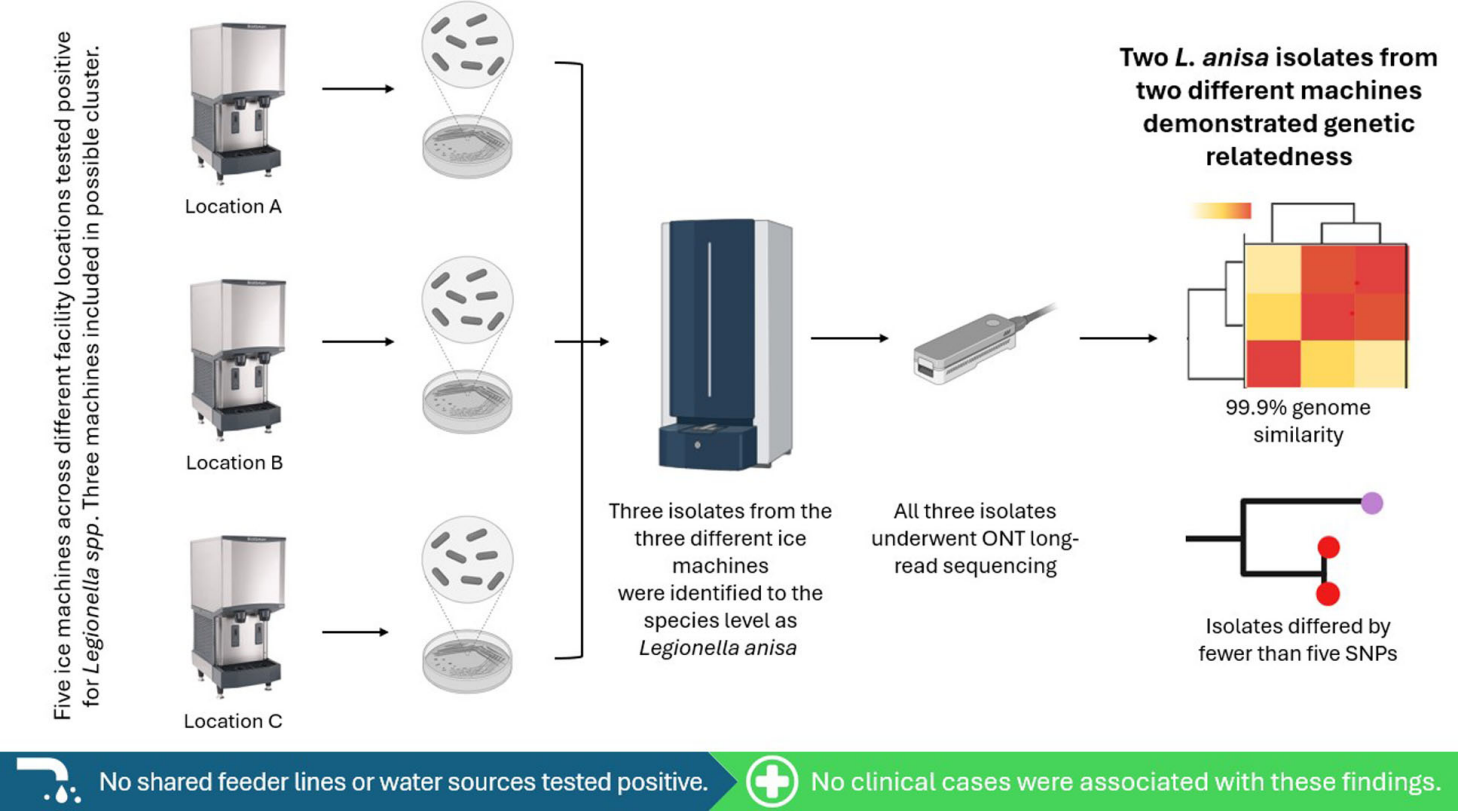# 308 Implementation of a Reflex Urine Culture Algorithm in a Pediatric Emergency Department: A Diagnostic Stewardship Assessment

**DOI:** 10.1017/ash.2026.10660

**Published:** 2026-06-23

**Authors:** Chin-Ting Wu, Guy Handley, Kim Cuong T Nguyen-Coleman, Noel Ellis, William Shropshire, Chia-Chi Chang, Nadim Ajami, Aaron G Richter, Linette Leadon, Matt Berkheiser, Samuel Shelburne, Amy Spallone

**Affiliations:** 1 The University of Texas MD Anderson Cancer Center; 2 MD Anderson Cancer Center; 3 UT MD Anderson Cancer Center; 4 University of Texas MD Anderson Cancer Center

## Abstract

**Background:**? Legionella poses a significant threat to immunocompromised oncology patients. To mitigate nosocomial legionellosis, healthcare systems often implement environmental surveillance. At MD Anderson Cancer Center, we combined a shared analytics platform with long-read whole genome sequencing (WGS) to enable detection of genetically related isolates from facility ice machines.? Methods:? A centralized dashboard (Power BI, Microsoft) was developed to monitor environmental Legionella surveillance across the institution and highlight areas with elevated positivity rates. Targeted environmental sampling was conducted to identify potential reservoirs. Given repeated isolation of L. anisa strains, a subset of isolates underwent WGS using the MinION platform (Oxford Nanopore Technologies). Genetic relatedness was assessed using MINTyper for single-nucleotide polymorphism (SNP) calling and Prokka with Roary for pan-genome analysis.? Results:? Environmental sampling of facility water sources revealed five positive Legionella samples from five separate ice machines, three of which grew L. anisa. No shared feeder lines or water sources tested positive. WGS performed by an infection control practitioner and revealed that two L. anisa isolates shared 99.9% genome similarity, differing by fewer than five SNPs, suggesting a common origin or transmission route (Figure 1). No clinical cases were associated with these findings during the investigation.? Conclusion:? A WGS based investigation powered by infection control practitioners without the use of a core genome sequencing facility uncovered potential transmission between geographically separated reservoirs that would have gone unrecognized through conventional methods. WGS also helped exclude a third ice machine from the suspected cluster. These findings led to the development of an updated institutional standard operating procedure (SOP), integrating data visualization and genomic analysis to enable earlier identification of related environmental sources and guide proactive interventions before clinical cases occur. This provides a framework for real-time implementation of WGS for Infection Control that can be deployed with minimal investment in nearly any clinical setting.

**Figure:**